# Functional and metabolic imaging of the right ventricle: short-term caloric restriction increases myocardial triglyceride content and decreases diastolic heart function

**DOI:** 10.1186/1532-429X-14-S1-P68

**Published:** 2012-02-01

**Authors:** Sebastiaan Hammer, RW Van Der Meer, Johannes A Romijn, Johannes W Smit, Albert de Roos, Hildo J Lamb

**Affiliations:** 1 Radiology, Leiden University Medical Centre, Leiden, Netherlands; 2 Endocrinology, Leiden University Medical Centre, Leiden, Netherlands

## Background

Caloric restriction increases plasma non-esterified fatty acid (NEFA) levels, and increases myocardial triglyceride content. It has been shown that elevated levels of NEFA’s in patients with metabolic disease (metabolic syndrome/ type 2 diabetes mellitus) are associated with a decrease in left ventricular function. However little is known about its effects on right ventricular function. Therefore, the purpose of the present study was to evaluate myocardial metabolic flexibility in relation to right ventricular diastolic function in healthy volunteers after short-term caloric restriction.

## Methods

Measures were performed in 14 healthy subjects before and after 3 days of caloric restriction based on Modifast diet (500 kcal/day) using cardiovascular magnetic resonance imaging (MRI). Myocardial triglyceride (TG) content was measured in the interventricular septum using navigator gated, proton MR spectroscopy. After water suppression, myocardial TG content was quantified as a percentage of the unsuppressed water signal. Right ventricular diastolic function was measured using MR velocity mapping across the tricuspid valve. Early (E) deceleration rate was quantified and regarded as representative for right heart diastolic function.

## Results

**Figure 1:**
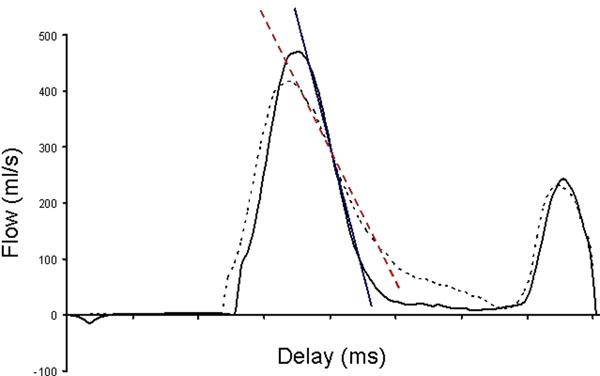
Typical trans-tricuspid flow curve and tangent lines of a subject before (solid lines) and after (dashed lines) caloric restriction, showing a decrease in early deceleration.

Caloric restriction increased plasma fatty acid levels from 0.53 ± 0.30 mmol/L at baseline to 1.14 ± 0.39mmol/L after caloric restriction. Concomitantly, myocardial TG content increased from 0.38 ± 0.19 % to 0.59 ± 0.24 % (P< 0.01). These changes were associated with a decrease in right ventricular diastolic function (E deceleration rate) from -1.79 ± 0.50 ml/sec2 x 10-3 to -1.25 ± 0.37 ml/sec2 x 10-3 (P< 0.01), example is shown in figure 1.

## Conclusions

Short-term caloric restriction in healthy volunteers increases myocardial triglyceride content and is associated with decreased right ventricular filling properties. The underlying pathophysiological mechanism may be involved in the development of global cardiac dysfunction in metabolic disease, such as obesity and diabetes type 2 related diseases.

## Funding

None.

